# Neuroprotective Effect of the Ginsenoside Rg1 on Cerebral Ischemic Injury In Vivo and In Vitro Is Mediated by PPAR*γ*-Regulated Antioxidative and Anti-Inflammatory Pathways

**DOI:** 10.1155/2017/7842082

**Published:** 2017-06-01

**Authors:** Yang Li, Yue Guan, Ying Wang, Chun-Lei Yu, Feng-Guo Zhai, Li-Xin Guan

**Affiliations:** ^1^Department of Orthopedics, Hongqi Hospital of Mudanjiang Medical University, Mudanjiang, Heilongjiang Province 157011, China; ^2^Department of Orthopedics, The First Affiliated Hospital of Harbin Medical University, Harbin, Heilongjiang Province 150001, China; ^3^Department of Endocrinology, Hongqi Hospital of Mudanjiang Medical University, Mudanjiang, Heilongjiang Province 157011, China; ^4^Department of Anatomy, Mudanjiang College of Medicine, Mudanjiang 157011, China; ^5^Department of Neurology, Hongqi Hospital of Mudanjiang Medical University, Mudanjiang, Heilongjiang Province 157011, China; ^6^Department of Pharmacology, Mudanjiang Medical University, Mudanjiang, Heilongjiang Province 157011, China

## Abstract

The ginsenoside Rg1 exerts a neuroprotective effect during cerebral ischemia/reperfusion injury. Rg1 has been previously reported to improve PPAR*γ* expression and signaling, consequently enhancing its regulatory processes. Due to PPAR*γ*'s role in the suppression of oxidative stress and inflammation, Rg1's PPAR*γ*-normalizing capacity may play a role in the observed neuroprotective action of Rg1 during ischemic brain injury. We utilized a middle cerebral artery ischemia/reperfusion injury model in rats in addition to an oxygen glucose deprivation model in cortical neurons to elucidate the mechanisms underlying the neuroprotective effects of Rg1. We found that Rg1 significantly increased PPAR*γ* expression and reduced multiple indicators of oxidative stress and inflammation. Ultimately, Rg1 treatment improved neurological function and diminished brain edema, indicating that Rg1 may exert its neuroprotective action on cerebral ischemia/reperfusion injury through the activation of PPAR*γ* signaling. In addition, the present findings suggested that Rg1 was a potent PPAR*γ* agonist in that it upregulated PPAR*γ* expression and was inhibited by GW9662, a selective PPAR*γ* antagonist. These findings expand our previous understanding of the molecular basis of the therapeutic action of Rg1 in cerebral ischemic injury, laying the ground work for expanded study and clinical optimization of the compound.

## 1. Introduction

Ischemic stroke is a central nervous system disease caused by transient or permanent reduction or interruption of arterial blood flow, typically caused by embolization or thrombosis. The condition accounts for approximately 80% of cerebrovascular disease and is often accompanied by severe physical and cognitive deficits. In addition to injury sustained by blood occlusion, injury can occur during the reperfusion process, adding a slew of additional complications [1]. Ultimately, the morbidity and mortality rates associated with cerebral ischemia remain high and are projected to climb with an aging population [2]. Currently, the leading treatments of ischemic cerebrovascular disease include the dissolution of thrombosis and intravascular therapies to restore blood supply to the brain. Thrombolytic therapy, though effective, is often limited by its narrow window of effectiveness and its reported increased risk of microvasculature injury [3]. A variety of alternative experimental neuroprotective strategies have struggled in clinical trials [4]. As such, the search for effective treatments against ischemic cerebrovascular disease and strategies to improve patient outcomes continues.

One emerging theory predicts that, due to the multifactorial pathogenesis of ischemic cerebrovascular disease [5], single target therapies will remain largely ineffective. Historically, the properties of traditional Chinese medicine have included increased tolerability (and subsequently reduced side effects), multiple-targeting capacity, and strong synergistic effects [6]. In previous studies, for example, traditional Chinese medicines like Rg1 have demonstrated efficacy in ischemic stroke prevention and treatment [7, 8]. The ginsenoside Rg1, extracted from ginseng root and stems, has shown antifatigue, antiaging, memory enhancing and neuroprotective characteristics. Recently, it was shown to significantly improve neurological function, reduce the infarct volume, and reduce the degree of cerebral edema after cerebral ischemia-reperfusion [9]. However, the mechanistic contributions of Rg1 specific pathways against the injury cascades of ischemic stroke remain to be clarified.

Cerebral ischemic injury is often the result of multifactorial pathogenic processes including inflammatory responses, oxidative stress responses, and apoptosis among others. Therefore, inhibition of the inflammatory response, reduction of oxidative stress, and cell death pathways have been highlighted as important strategies for prevention and treatment of ischemic cerebrovascular disease. Peroxisome proliferator activated receptors (PPARs), a class of ligand-activated nuclear transcription factors involved in the regulation of both inflammatory processes and oxidative stress, have been previously evaluated in cerebral ischemic injury, where they are reported to play a regulatory role in the inhibition of the inflammatory response and oxidative stress responses that follow injury. Particularly, these studies have revealed PPAR*γ* agonists as effective therapeutic targets of ischemic cerebrovascular disease, reducing infarct volume and improving neurological function [10, 11].

A study in 2010 reported that Rg1 treatment could improve the expression of PPAR*γ* and lipid metabolism in the treatment of Type 2 diabetes [12]. More recently, the antiapoptotic and anti-inflammatory capacity of a downstream PPAR*γ* effector has been reported in a rat model of cerebral ischemic injury [13]. Due to the involvement of PPAR*γ* in neuroinflammatory and oxidative processes, as well as the previously reported neuroprotective properties of Rg1, we sought to investigate whether Rg1 expressly affects PPAR*γ* signaling in cerebral ischemic injury. Using a rat focal cerebral ischemia model (MCAO) and rat cerebral cortical neuron ischemic injury model (OGD), this study aimed to answer whether the ginsenoside Rg1 could activate PPAR*γ*-mediated anti-inflammatory and antioxidative effects, establishing a mechanistic groundwork for Rg1 in the therapeutic scope of ischemic cerebrovascular disease.

## 2. Materials and Methods

### 2.1. Antibodies and Reagents

In this study, Rg1 (HPLC purity > 98%) and GW9662 (HPLC purity > 98%) were procured from Sigma-Aldrich (cat #68317, cat #M6191). MPO (cat #A044), CAT (cat #A007-1), and SOD (cat #A001-3) detection kits were purchased from Jiancheng Bioengineering Institute and IL-6 (cat #ERC003) and TNF-*α* (cat #ERC102a) ELISA Kits from Neobioscience Technology Company. PPAR*γ*, NF-*κ*B65, and *β*-actin primary antibodies were purchased from Santa Cruz Biotechnology (USA) and Tris-HCl, SDS, Triton X-100, trypsinase, and glycocine from Ameresco (USA). All chemicals and reagents used were of analytical grade.

### 2.2. Animals

Sixty adult male Sprague-Dawley rats (270–320 g) were obtained from the Institute of Beijing Weitonglihua Experimental Animals, Beijing, China (License number 2006-0009). Animals were housed at 22 ± 2°C in relative humidity of 50 ± 10% with a 12-hour light/dark cycle and free access to chow and water. The animal care and experimental protocols in this study were evaluated and found to be in accordance with the Guidance Suggestions for the Care and Use of Laboratory Animals issued by the Ministry of Science and Technology of the People's Republic of China.

### 2.3. Rat Middle Cerebral Artery Occlusion (MCAO) Model

Focal cerebral ischemia/reperfusion injury was induced in the experimental group by occlusion of the middle cerebral artery (MCAO) using a previously described method [14].

Briefly, anesthesia was administered by intraperitoneal injection of 350 mg/kg chloral hydrate, after which the right common carotid artery, internal carotid artery, and external carotid artery were surgically exposed. A 4-0 monofilament nylon suture (Beijing Sunbio Biotech, Beijing, China) with a rounded tip was inserted into the internal carotid artery through the external carotid arterial stump and gently advanced to occlude the middle cerebral artery. Ninety minutes after the onset of middle cerebral arterial occlusion, the suture was removed to restore blood flow for the following 24 hours. Rats in the sham-surgery group were surgically manipulated in the same way, with the exception of middle cerebral arterial occlusion. The rectal temperature was monitored and maintained at 37.0 ± 0.5°C with a heating pad throughout the surgical procedure.

### 2.4. Cell Culture and Oxygen Glucose Deprivation (OGD) Model

Primary cultured cortical neurons were isolated as previously described with slight modifications [15]. Briefly, cerebral cortex was obtained from the fetus of pregnant Sprague-Dawley rats (embryonic day 16). Cerebral cortices were placed into the cold D-Hank's solution after careful removal of meningeal tissue. The cortices were minced and digested with 1.25 g/L trypsin (Sigma Chemical Co., USA) for 30 minutes at 37°C. Next, the cortical tissues were dispersed gently by pipetting and the cell suspension was filtered through a nylon mesh. The cells were plated on poly-lysine-coated dishes at a density of 1 × 10^9^/L. Plates or flasks were maintained in DMEM medium supplemented with 10% (v/v) fetal bovine serum (Hyclone Corp., USA), 10% (v/v) horse serum (Hyclone Corp., USA), penicillin G (100 U/ml), and streptomycin (0.1 g/L). Cultures were kept at 37°C in a 5% CO_2_ atmosphere. Twenty-four hours after plating, the culture medium was replaced with DMEM medium containing 10% (v/v) horse serum, 1% (v/v) N2 supplement (Invitrogen Corp., USA), 100 U/ml penicillin G, and 0.1 g/L streptomycin. Seventy-two hours after plating, cells were incubated with 2.25 mg/L cytosine arabinoside (HuaLian Pharmaceutical Ltd., Shanghai, China) for 48 hours to inhibit the growth of nonneuronal cells. After nonneural growth inhibition, cell culture medium was changed twice a week. The experiments presented in this study were performed on neurons grown for 10–14 days in vitro. Immunochemical staining with antineuron-specific enolase (Boster Biological Technology Ltd., Wuhan, China) revealed that this culture protocol yielded cell cultures containing about 85% neurons.

An oxygen glucose deprivation (OGD) model was established on cortical cell cultures as previously described [16]. Briefly, 10–14 days after culture, the primary cortical neurons were washed with glucose-free DMEM (Gibco, USA) and placed in an anaerobic chamber at 5% CO_2_ and 95% N_2_ at 37°C. Oxygen deprivation was terminated after 90 min by replacing the glucose-free DMEM medium with the original medium, at which point neurons were incubated in a chamber at 95% O_2_ and 5% CO_2_ at 37°C for 24 h. All drugs and agents were added to the culture medium immediately following the 90 min OGD session. Cells in the control group were treated without the OGD exposure.

### 2.5. Drugs Treatment

In in vivo experiments, rats were divided randomly into the following groups: sham-surgery (surgical control), MCAO treated with saline (injury model), MCAO treated with 30 mg/kg Rg1, MCAO treated with 60 mg/kg Rg1, and MCAO treated with Rg1 (60 mg/kg) and GW9662 (10 mg/kg). Different doses of drugs were administered intraperitoneally at 90 min (at the time of MCAO termination/reperfusion) and 6 hrs after reperfusion. The control and injury model groups were treated with equal volumes of saline. Analyses were performed from 24 hours after ischemia-reperfusion.

In in vitro experiments, the primary cortical neurons were cultured for 10–14 days prior to being randomly divided into following groups: control group (control), OGD model group, OGD model treated with 30 *μ*mol/L Rg1 (Rg1-Low), OGD model treated with 60 *μ*mol/L (Rg1-High), and OGD model treated with Rg1 (60 *μ*mol/L) and GW9662 (10 *μ*mol/L). Drugs were added to the medium 90 minutes after induction of OGD. Cell analyses were performed 24 hours after the onset of cellular hypoxia (ischemia-reperfusion model).

### 2.6. Evaluation of Neurological Function

A neurological function evaluation was performed using Longa's method [17]. Evaluations were performed by a single investigator, blinded to the experimental groups, 1 and 3 days after injury. Neurological capacity was scored on a 5-point scale according to the following indications: no neurological deficit = 0, failure to extend left paw fully = 1, circling to left = 2, falling to left = 3, not walking spontaneously, and having depressed levels of consciousness = 4. Following surgery, rats with a neurological score between 1 and 3 were selected for the study, and rats with a score of <1 were considered to have unsuccessful MCAO induction.

### 2.7. Quantification of Brain Water Content

The swelling of ischemic brain tissue was evaluated by examination of brain water content according to the wet-dry method [18]. In brief, rats were decapitated under deep anesthesia with 10% chloral hydrate after 24 h of reperfusion. Brains were immediately acquired and a neutral filter paper was used to absorb and remove blood stains from the brain. The ipsilateral and contralateral cortical hemispheres were dissected and the wet weight of the tissue was determined by an electronic scale (wet weight). Subsequently, tissues were dried overnight in a desiccating oven kept at 105°C after which the dry weight was obtained. Finally, the brain water content was calculated according to the following formula: brain water content (%) = {(wet  weight − dry  weight)/wet  weight} × 100%.

### 2.8. Detection of MPO, SOD Activity, and CAT Content

Total protein was isolated from cortical tissue and cell homogenates prepared in ice cold saline. MPO, SOD activity, and CAT content were measured using commercially available detection kits (Nanjing Jiancheng Bioengineering Institute, Nanjing, China), according to the manufacturers' instructions.

### 2.9. Enzyme-Linked Immunosorbent Assays (ELISAs)

Measurement of inflammatory markers was performed by enzyme-linked immunosorbent assay (ELISA). The secreted levels of interleukin-6 (IL-6) and tumor necrosis factor-*α* (TNF-*α*) in the ischemic brain were measured by commercially available ELISA kits (Neobioscience Technology Company, Shenzhen, China) according to the manufacturer's instructions. The absorbance was measured at 450 nm on a microplate reader (Molecular Devices Corp., Sunnyvale, CA, USA).

### 2.10. Western Blot Analysis

Protein from cerebral ischemic cortex or cortical neurons was collected and diluted to a concentration of 0.5 mg protein/mL for the measurement of PPAR*γ* and NF-*κ*B 65. Equal amounts of protein were electrophoresed through a reducing sodium dodecyl sulfate polyacrylamide gel and electroblotted onto a polyvinylidene difluoride membrane. After incubation in blocking buffer (5% nonfat dried milk, phosphate-buffered saline, and 0.1% Tween-20) at room temperature for 1 hour, membranes were incubated with mouse anti-rat PPAR*γ* monoclonal antibody (1 : 500; Santa Cruz Biotechnology, Santa Cruz, CA, USA), mouse anti-rat NF-*κ*B 65 monoclonal antibody (1 : 500; Santa Cruz Biotechnology), or rabbit anti-*β*-actin polyclonal antibody (1 : 3,000; Santa Cruz Biotechnology) overnight at 4°C. Protein levels were detected with goat anti-rabbit or anti-mouse IgG-horseradish peroxidase-linked secondary antibodies (1 : 2,000; Santa Cruz Biotechnology) at room temperature for 1 hour and developed with Super ECL Plus detection reagent (Applygen Technologies, Beijing, China). Films were scanned and the intensities of immunoblot bands were quantified by densitometry using image analysis software (Image Master Total Lab version 1.00; Amersham Pharmacia Biotech, Tokyo, Japan). The band absorbance values were calculated as a ratio of PPAR*γ*/*β*-actin or NF-*κ*B 65/*β*-actin.

### 2.11. Statistical Analysis

Analysis of variance was performed with SPSS 13.0 statistical software (SPSS, Inc., Chicago, IL, USA). The results are expressed as mean ± SD. A value of *P* < 0.05 was considered a statistically significant difference between groups.

## 3. Results

### 3.1. Effects of Rg1 on Neurological Deficits of Cerebral Ischemic Rats

After successful induction of focal cerebral ischemia/reperfusion injury by the MCAO method, we evaluated the effect of Rg1 on neurological deficits via Longa's method. The results showed that, in the sham group, rats appeared to have no symptoms of neurological impairment. In contrast, rats in the injury model group showed significantly increased neurological deficit scores compared to the control group (*P* < 0.01). However, administration of 60 mg/kg Rg1 decreased the neurological deficit scores compared to the injury model group (*P* < 0.05, Table 1). These data indicated that treatment with Rg1 significantly ameliorated the observed neurological impairment occurring after cerebral ischemic injury in rats.

### 3.2. Effects of Rg1 on Cerebral Edema in Cerebral Ischemic Rats

Postischemic brain edema, as a secondary indicator of the extent of cerebral ischemia was evaluated. Brain water content was remarkably increased in the injury model group compared with control animals (*P* < 0.01, Table 1). In contrast, animals treated with Rg1 60 mg/kg demonstrated a significant reduction in observed brain water content in comparison with the untreated injury group (*P* < 0.05, Table 1). These findings echo the neuroprotective effects of Rg1 in cerebral ischemia demonstrated in the first experiment.

### 3.3. Effect of Rg1 on Inflammatory and Oxidative Markers in Cerebral Ischemic Rats

Myeloperoxidase (MPO) is an enzyme secreted during inflammatory processes and is commonly used as a marker of tissue infiltration of inflammatory cells. Compared to the control group, MPO activity was significantly increased in the injury model group (*P* < 0.01). In contrast, the measured levels of the antioxidants superoxide dismutase (SOD) and catalase (CAT) were significantly decreased in the injury model group compared to controls (*P* < 0.01, *P* < 0.05, resp.). As shown in Table 2, we observed that treatment with Rg1 significantly decreased elevated MPO activity (*P* < 0.05) and normalized the injury-diminished levels of SOD and CAT compared with the untreated injury group (*P* < 0.05). Collectively, these results indicated that Rg1 could significantly alleviate the inflammation and oxidative stress response which occurs after cerebral ischemic injury in rats.

### 3.4. Effect of Rg1 on Oxidative Stress Markers in OGD Rat Cortical Neurons

SOD activity and CAT levels were evaluated in a model of oxygen glucose deprivation (OGD) which was selected as a secondary assessment tool due to their identification as neuron-specific correlates of cerebral ischemic injury [19]. Similar to the cerebral ischemic injury model, SOD and CAT levels were significantly lowered in cortical neurons by OGD injury compared with the control group (*P* < 0.01, *P* < 0.05, resp.). Conversely, SOD activity and CAT levels were significantly elevated by 60 *μ*mol/L treatment with Rg1 compared with untreated OGD neurons (*P* < 0.05, Table 3).

### 3.5. Effect of Rg1 on Inflammatory Cytokines in Cerebral Ischemic Rats

It has been well documented that inflammatory-associated cytokines such as IL-6 and TNF-*α* play a chief role in inflammatory activation upon cerebral ischemia-reperfusion injury. In this study, ELISA assays were performed to measure the content of IL-6 and TNF-*α* detected after injury and treatment. As shown in Table 4, the presence of IL-6 and TNF-*α* was significantly increased in MCAO-injured cortical brain tissue compared with the control group (*P* < 0.01). However, these elevated levels of IL-6 and TNF-*α* were significantly diminished by administration of Rg1 compared to untreated injured animals (*P* < 0.05). These results demonstrated that Rg1 can significantly relieve the inflammatory response occurring after cerebral ischemic injury in rats.

### 3.6. Effect of Rg1 on Inflammatory Cytokines in OGD Rat Cortical Neurons

An evaluation of the inflammatory cytokines was undertaken in OGD-cortical neurons to confirm the patterns observed in the MCAO injury model. As shown in Table 5, IL-6 and TNF-*α* were all significantly increased in the cortical neurons injured by OGD compared with the control group (*P* < 0.01). However, the levels of IL-6 and TNF-*α* were all significantly decreased by treatment with Rg1 compared to untreated OGD neurons (*P* < 0.05). The results provided further evidence that Rg1 can significantly relieve the inflammatory response occurring after hypoxic injury in cortical neurons.

### 3.7. Effect of Rg1 on PPAR*γ* and NF-*κ*B 65 Expression in Cerebral Ischemic Rats

**Figure 1:**
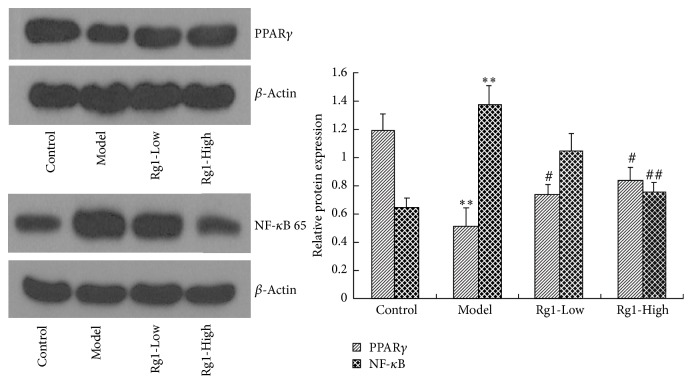
Effect of Rg1 on the protein expression of PPAR*γ* and NF-*κ*B 65 in brain tissue of rats. ^*∗∗*^*P* < 0.01 versus control group; ^##^*P* < 0.01 and ^#^*P* < 0.05 versus model group.

To answer whether Rg1 could normalize PPAR*γ* expression in cerebral ischemia (as observed in a model of Type 2 diabetes) Western blot analysis was used to detect the protein levels of PPAR*γ* and NF-*κ*B 65, a marker of inflammatory and immune responses, in rat brain tissue. Compared with the sham-surgery group, 24 hours after cerebral ischemia, PPAR*γ* protein levels were significantly decreased (*P* < 0.01), while NF-*κ*B 65 protein levels were significantly increased (*P* < 0.01) in the MCAO injury group. As shown in Figure 1, we further observed that treatment with Rg1 significantly normalized PPAR*γ* protein levels (*P* < 0.05) and attenuated NF-*κ*B 65 protein levels compared with untreated, injured animals (*P* < 0.05). These findings illustrate the therapeutic action of Rg1 on inflammatory response and confirm the Rg1-mediated elevation of PPAR*γ* observed in previous studies.

### 3.8. Effect of Rg1 on PPAR*γ* and NF-*κ*B 65 Expression in OGD Rat Cortical Neurons

**Figure 2:**
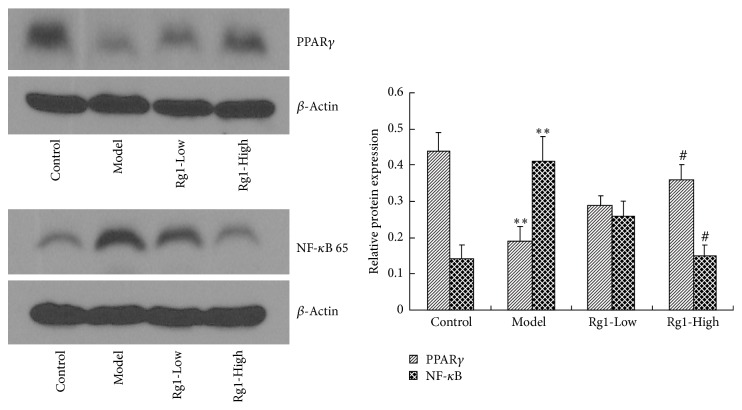
Effect of Rg1 on the protein expression of PPAR*γ* and NF-*κ*B 65 in the cortical neurons of rats. ^*∗∗*^*P* < 0.01, versus control group; ^#^*P* < 0.05 versus model group.

Finally, the effects of Rg1 on PPAR*γ* and NF-*κ*B 65 expression were evaluated in a secondary, extracorporeal model of neural hypoxic injury. Compared with the control group, 24 hours after OGD injury, PPAR*γ* protein levels were significantly decreased (*P* < 0.01), while NF-*κ*B 65 protein levels were significantly increased (*P* < 0.01). As shown in Figure 2, we observed that treatment with 60 *μ*mol/L Rg1 significantly increased PPAR*γ* protein levels (*P* < 0.05) and decreased NF-*κ*B 65 protein compared with the untreated OGD neurons (*P* < 0.05). Once again, these results confirm the anti-inflammatory action of Rg1 and the regulatory capacity of the compound on PPAR*γ* in neurons.

### 3.9. Rg1 Induced PPAR*γ* Expression and Was Inhibited by GW9662 in Cerebral Ischemic Rats and in OGD Rat Cortical Neurons

**Figure 3:**
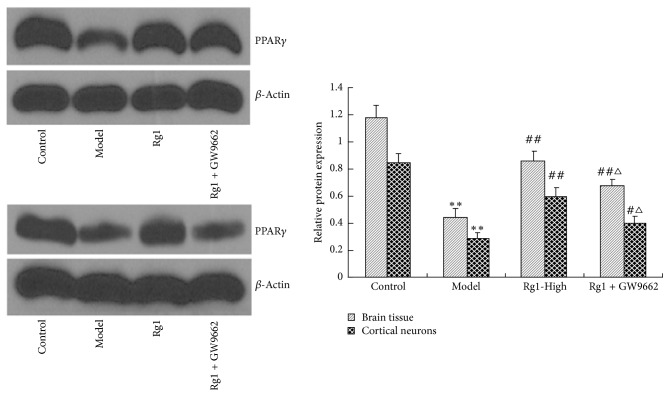
Rg1 induced PPAR*γ* expression and inhibited by GW9662 in cerebral ischemic rats and in OGD rat cortical neurons. ^*∗∗*^*P* < 0.01 versus control group; ^##^*P* < 0.01 and ^#^*P* < 0.05 versus model group; ^△^*P* < 0.05 versus Rg1-High group.

In order to further demonstrate the PPAR*γ*-dependent mechanism in the neuroprotection of Rg1, we investigated the effects of Rg1 cotreatment with GW9662 on PPAR*γ* expression in cerebral ischemic rats and in OGD rat cortical neurons. As shown in Figure 3, the results showed that the expression of PPAR*γ* significantly increased after Rg1 treatment in cerebral ischemic rats and in OGD rat cortical neurons (*P* < 0.01). The upregulating of PPAR*γ* induced by Rg1 was inhibited by GW9662 (*P* < 0.05), an antagonist of PPAR*γ*. These suggested that Rg1 was a potent agent to promote PPAR*γ* expression.

## 4. Discussion

Though several therapies are available for the treatment of cerebral ischemia/reperfusion injury, they have severe limitations including toxicity, side effects, and singularity of targets. Due to their increased tolerability, synergism, and so on, many traditional Chinese medications have been evaluated as alternatives in various neurological diseases, including cerebral ischemia. The ginsenoside Rg1 has demonstrated neuroprotective capacity in cerebral ischemia [13, 20], though its molecular underpinnings have not been thoroughly understood. A report in 2010 showed that Rg1 could increase the expression of PPAR*γ* mRNA, encoding PPAR*γ* receptors involved in the regulation of a barrage of biological processes including lipid metabolism and the regulation of inflammatory and oxidative responses [12]. Additional evidence has implicated PPAR*γ* signaling as a contributor to the neurodegenerative processes of cerebral ischemic injury. For example, a PPAR*γ* inducible haemoxygenase, which has demonstrated sensitivity to oxidative stress and protective properties during oxidative tissue damage [21], was activated by Rg1 in a rat model of cerebral ischemic injury [13]. Further, the influence of PPAR*γ* on proinflammatory cytokines has also been demonstrated in rat MCAO models [22]. Due to the role that inflammatory and oxidative processes play in the etiology of cerebral ischemia, we hypothesized that Rg1 neuroprotection may occur through recruitment of PPAR*γ* signaling in the ischemic brain. Present data revealed that Rg1 markedly increase the expression of PPAR*γ* in cerebral ischemic rats and in OGD rat cortical neurons, and then we also found that the selective PPAR*γ* antagonist GW9662 can decrease the expression of PPAR*γ*, suggesting that Rg1 might be a potent agonist of PPAR*γ*.

Using a MCAO rat model of cerebral ischemia/reperfusion injury, we observed that Rg1 (administered at 60 mg/kg) effectively diminished neurological deficits and brain edema-hallmarks of cerebral ischemic injury. Ultimately, these results echo the results of previous studies which have indicated the effectiveness of Rg1 as a neuroprotectant in various models of cerebral ischemic injury, including reduced infarct volume and neurological deficit [13, 20, 23].

In the investigation of the molecular drivers of Rg1's neuroprotective capacity, we focused on inflammatory and oxidative stress pathways due to their demonstrated elevation in ischemic injury response and their proposed influence downstream of PPARy signaling. For example, PPAR*γ* response elements like heme oxygenase-1 have been shown to aid in the inhibition of apoptosis and inflammation [24–26]. Studies of PPAR*γ* agonists have further supported the pathway's role in ischemic injury responses [27, 28]. Alternatively, targeted inhibition of PPAR*γ* has demonstrated that PPAR*γ* is necessary to facilitate the neuroinflammatory protection observed during cerebral ischemia [22].

Here, we found that the 60 mg/kg dose of Rg1 was able to normalize increased expression of the inflammatory marker MPO. Simultaneously, Rg1 was shown to normalize the diminished expression of antioxidant enzymes SOD and CAT. These observations were subsequently confirmed in vitro in rat cortical neurons. This demonstrates the multifactorial nature of Rg1 and more directly implicates the biological pathways by which the compound acts as a neuroprotectant in cerebral ischemia. To expand the investigation of Rg1's role in PPAR*γ*-mediated inflammatory response, we examined the expression of the intersectional inflammatory cytokines TNF alpha and IL-6. The PPAR*γ* agonist rosiglitazone has been shown to inhibit TNF alpha production in microglia in progressive neurodegeneration models [29]. Interestingly, TNF alpha has been linked to increased production of mitochondrial superoxides in oligodendrocyte progenitors [30], implicating the cytokine in the inhibition of PPAR*γ*'s antioxidative pathways as well. Here we observed that Rg1 treatment reduced TNF alpha expression, supporting our earlier observations of normalized expression of inflammatory cytokines.

Rg1 treatment demonstrated a similar effect on the expression of the cytokine IL-6. Previously, a PPAR*γ* agonist in a traumatic brain injury model was shown to decrease the expression of IL-6, supporting that PPAR*γ* plays a regulatory role in the expression of brain injury related inflammation [31]. Interestingly, an IL-6 gene variant has been identified as a predictor of ischemic stroke in an epidemiological study [32].

Finally, NF-*κ*B, a master regulator of cell survival and inflammation, was found to be diminished by Rg1. NF-*κ*B activation has been previously demonstrated in models of cerebral ischemia, where it demonstrates sensitivity to reactive oxygen species as well as several inflammatory mediators [33]. This and other studies point at a likely complex interaction of Rg1 and PPAR*γ* in cerebral ischemic injury, as many downstream effectors involved in inflammation response have demonstrated cell survival and/or oxidative stress regulation as well. Additionally, aging studies have identified that the proteolytic properties of PPAR*γ* may contribute to progressive loss of cellular metabolic capacity and may contribute to loss of microvascular integrity [2], explaining the comorbidity between cerebral ischemia and neurodegenerative diseases as well as expanding the urgency and impact of therapies for cerebral ischemic injury, which may set in motion the basis for various neurological risks after injury [34].

In this study, in vitro examinations of OGD-cortical neurons were used to supplement the results of the experimental rat model, allowing a more monocellular scope of investigation. These experiments agreed with the effects of Rg1 on PPAR*γ*-mediated oxidative and inflammatory pathways, bolstering the interaction of Rg1 on PPAR*γ* pathways in cortical neurons. Other in vitro studies of Rg1 have similarly identified an antioxidative role for Rg1, improving cell viability in hypoxic and ischemic models [35–37]. And while a more direct study of the molecular interaction of Rg1 and the PPAR*γ* axis is needed, this study provides a more definitive scope of biological actions by which to deepen our investigations and evaluate the optimization of the compound. Consequently, Rg1 may also be experimentally investigated in other diseases brought about by dysregulation of inflammation and or oxidative responses.

## 5. Conclusions

In this study, an in vivo and in vitro model of cerebral ischemia was used to answer whether the neuroprotective effects of the traditional Chinese medicine Rg1 could be attributed to PPAR*γ*-mediated regulation of cerebral inflammation and oxidative stress. We report that the compound, administered at high doses, was capable of attenuating the activation of various oxidative species through the upregulation of endogenous antioxidants. Further, the compound diminished activation of inflammatory cytokines and the NF-*κ*B inflammatory signal. These observations at least partially clarify the molecular drivers of the neuroprotective effect of Rg1 in cerebral ischemia/reperfusion injury, laying the groundwork for more detailed examination and optimization of the treatment in future, preclinical and clinical studies.

## Figures and Tables

**Table 1:** Effects of Rg1 on neurological deficit score and brain edema ($\bar{x}\pm S,\;\;n=10$).

Group	Neurological deficit score (score)	Brain water content (g)
Control	0	77.88 ± 1.35
Model	2.10 ± 0.69^*∗∗*^	82.35 ± 2.56^*∗∗*^
Rg130 mg/kg	1.80 ± 0.37	80.27 ± 2.11
Rg160 mg/kg	1.40 ± 0.47^#^	79.36 ± 1.65^#^

^*∗∗*^ *P* < 0.01 versus control group; ^#^*P* < 0.05 versus model group.

**Table 2:** Effect of Rg1 on MPO, SOD activity, and CAT content in brain tissue of rats ($\bar{x}\pm S,\;\;n=6$).

Group	MPO (U·g^−1^)	SOD (U/mg·pro)	CAT (U/mg·pro)
Control	0.20 ± 0.03	133.50 ± 18.07	22.17 ± 2.91
Model	0.33 ± 0.04^*∗∗*^	95.83 ± 16.43^*∗∗*^	15.17 ± 2.54^*∗*^
Rg130 mg/kg	0.30 ± 0.03^#^	100.67 ± 12.53	16.83 ± 2.48
Rg160 mg/kg	0.26 ± 0.03^#^	120.17 ± 15.29^#^	20.33 ± 2.69^#^

^*∗∗*^ *P* < 0.01, ^*∗*^*P* < 0.05 versus control group; ^#^*P* < 0.05 versus model group.

**Table 3:** Effect of Rg1 on antioxidants SOD and CAT in OGD rat cortical neurons ($\bar{x}\pm S,\;\;n=6$).

Group	SOD (U/mg·pro)	CAT (U/mg·pro)
Control	28.83 ± 4.18	10.05 ± 1.16
Model	19.17 ± 3.13^*∗∗*^	8.03 ± 1.09^*∗*^
Rg130 *µ*mol/L	20.92 ± 3.06	8.93 ± 1.02
Rg160 *µ*mol/L	25.31 ± 2.87^#^	9.82 ± 1.07^#^

^*∗∗*^ *P* < 0.01, ^*∗*^*P* < 0.05 versus control group; ^#^*P* < 0.05 versus model group

**Table 4:** Effect of Rg1 on the content of TNF-*α* and IL-6 in brain tissue of rats ($\bar{x}\pm S,\;\;n=6$).

Group	TNF-*α* (ng/mg)	IL-6 (pg/mg)
Control	0.71 ± 0.12	95.17 ± 11.13
Model	1.11 ± 0.14^*∗∗*^	131.33 ± 19.15^*∗∗*^
Rg130 mg/kg	1.00 ± 0.13	108.83 ± 14.15^#^
Rg160 mg/kg	0.89 ± 0.12^#^	101.50 ± 12.33^#^

^*∗∗*^ *P* < 0.01, versus control group; ^#^*P* < 0.05 versus model group.

**Table 5:** Effect of Rg1 on the content of TNF-*α* and IL-6 in the cortical neuron of rats ($\bar{x}\pm S,\;\;n=6$).

Group	TNF-*α* (pg/mg)	IL-6 (pg/mg)
Control	23.96 ± 4.25	9.55 ± 1.17
Model	37.12 ± 6.28^*∗∗*^	15.95 ± 2.37^*∗∗*^
Rg130 *µ*mol/L	32.2 ± 5.32	13.54 ± 1.89^#^
Rg160 *µ*mol/L	30.27 ± 4.35^#^	12.56 ± 1.14^#^

^*∗∗*^ *P* < 0.01, versus control group; ^#^*P* < 0.05 versus model group.
